# Supplementary material for: Identification of photoperiod-induced specific miRNAs in the adrenal glands of Sunite sheep (Ovis aries)
Source: Front Vet Sci. 2022 Jul 22;9:888207. doi: 10.3389/fvets.2022.888207 (PMC9354845; doi:10.3389/fvets.2022.888207)
Supplement: Supplementary file 1 [file Data_Sheet_1.ZIP › Supplementary file/miRDeep2 file.zip!/miRDeep2 file/Novel_Structure/SPLP42A3/Novel_277.pdf]

Provisional ID	:	NC_019470.2_23779
Score total	:	1.8
Score for star read(s)	:	-1.3
Score for read counts	:	0
Score for mfe	:	1.5
Score for randfold	:	1.6
Score for cons. seed	:	
Total read count	:	20
Mature read count	:	20
Loop read count	:	0
Star read count	:	0

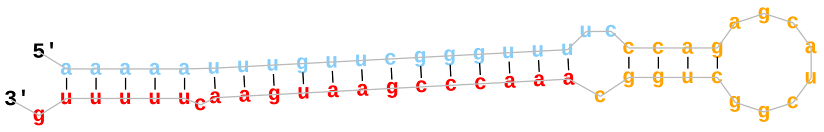

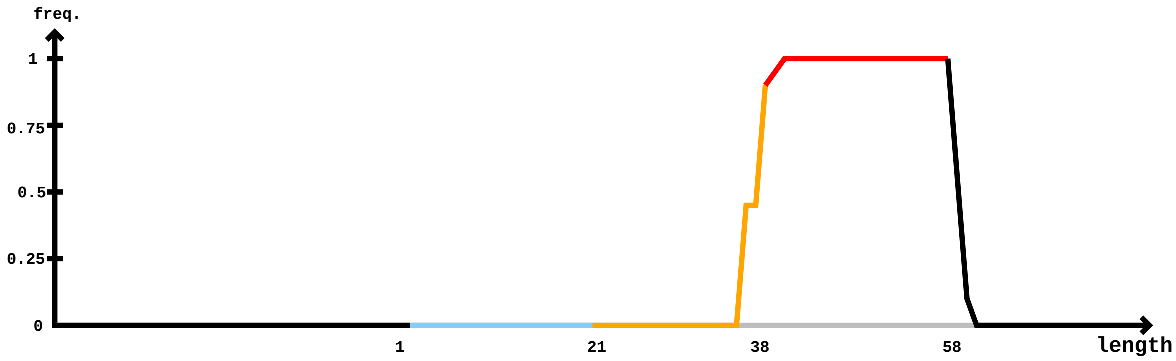

Star　　　　　　Mature

```
5'- guguugaucuaacacacuauaucucagguuggucaaaaaauuuguucggguuuucccagagcaucggcuggcaaacccgaaugaacuuuuuggucaacucacuacucuggca   -3'    exp
    ((((((...))))))...........(((((((((((((.(((((((((((((..((((........)))).))))))))))))).)))))))))))))).............    reads      mm         sample
    ......................................................................gcaaacccgaaugaacuuuuug....................    2          0          seq
    ......................................................................gAaaacccgaaugaacuuuuug....................    6          1          seq
    ......................................................................gAaaacccgaaugaacuuuuugg...................    1          1          seq
    ........................................................................aaacccgaaugaacuuuuuA....................    7          1          seq
    ........................................................................aaaccAgaaugaacuuuuug....................    1          1          seq
    ........................................................................aaacccAaaugaacuuuuuggu..................    1          1          seq
    .........................................................................aacccgaaugaacuuuuuggu..................    1          0          seq
    ..........................................................................acccgaaugaacuuuuugg...................    1          0          seq
```
